# 

**Published:** 1890-11

**Authors:** 


### CONTENTS—NOVEMBER, 1890.—VOL. VI., NO. 5.

ORIGINAL ARTICLES— PAGE.

Preliminary Capsulotomy in the Extraction of Cataract.—T. J. Tyner, M. D. .... 189
Malarial Hæmaturia.—H. A. Tutwiler, M. D. .... 192
Sudden Death During Labor. Report of a Case.—L. H. Hardy, M. D. .... 197
Ice Water in Cholera Infantum.—E. McD. Bridgeford, M. D. .... 198
Traumatic Erysipelas Aborted.—D. Du Pre, M. D. .... 200

CORRESPONDENCE—

Unjust Treatment of Physicians by the State. Letter from Dr. Johnston .... 203

SOCIETY NOTES—

Proceedings Terrell Medical Society. Important action .... 205
North Texas Medical Association, the Coming Meeting .... 207
Resolutions by the Galveston Club. Resolutions by the Travis County Medical Society, and also by the West Texas Medical Society .... 209

ITEMS OF INTEREST—

Therapeutics, etc .... 210

EDITORIAL—

Injustice to Doctors .... 213
One More Effort .... 215

BOOK NOTICES—

A New Medical Dictionary .... 217
An Encyclopedia of Practical Medicine, Based on Bacteriology, etc .... 217

MEDICAL NEWS AND MISCELLANY—

Numerous Items of Interest .... 221

PUBLISHER'S NOTES—

Containing Valuable Information .... 229

